# Two steps to space for numbers

**DOI:** 10.3389/fpsyg.2015.00612

**Published:** 2015-05-12

**Authors:** Martin H. Fischer, Samuel Shaki

**Affiliations:** ^1^Division of Cognitive Sciences, Department of Psychology, University of PotsdamPotsdam, Germany; ^2^Department of Behavioral Science, Ariel UniversityAriel, Israel

**Keywords:** spatial-nunmerical association, SNARC, mental number line, numerical cognition, spatial cognition

The study of spatial-numerical associations (SNAs) is an active research project that was triggered by a landmark publication reporting several simple reaction time experiments: Adults classified visually presented numbers according to their parity by using left and right response keys (Dehaene et al., 1993). The main finding was that small numbers, such as 1 or 2, were classified faster on the left side and larger numbers, such as 8 or 9, were classified faster on the right side. This specific instance of a SNA has been replicated and extended in numerous studies (recent review by Fischer and Shaki, 2014). The original interpretation of the effect assumed a “spill-over” from reading habits into the number domain but subsequent work has pushed back the time line to preschoolers, infants, and even neonates (for recent review, see Patro et al., 2014). Our own work (e.g., Shaki et al., 2009; Fischer and Shaki, 2015) confirmed that reading habits contribute to the direction and strength of SNAs but has also indicated that they are not the only and not even the strongest determinant (e.g., Fischer et al., 2010). In the following paragraphs we propose a processing principle for SNAs and describe two successive steps by which the mapping of numbers onto space might occur.

Our proposed processing principle is that *spatial mapping is an integral part of semantic number processing*. This is evident from the ubiquity of SNAs: They have been reported with various stimulus formats, in many different tasks, and while studying a wide range of responses (for recent review, see Fischer and Shaki, 2014). SNAs modulate the cortical region underlying semantic number processing (i.e., bilateral hIPS; Cutini et al., 2012). Moreover, the association between numbers and space is bi-directional: numerical magnitude can serve as a spatial cue and vice versa (Stoianov et al., 2008; Shaki and Fischer, 2014a). Most studies of SNAs have used centrally presented numbers in combination with spatial responses, which may have encouraged participants to use spatial number mapping strategies (Fischer, 2006). However, today it is clear that the very appearance of numerical stimuli is enough for SNAs to appear, even when removing, in healthy adults (cf. Zorzi et al., 2002), spatial features from *both* stimuli and responses (Fischer and Shaki, 2015; Ranzini et al., 2015). Evidence for such a purely conceptual link between numbers and space was even found in Hebrew speakers, thus requiring correction of our earlier claim of the need for consistency of directional processing habits across stimulus domains (Shaki et al., 2009; Shaki and Fischer, 2012, 2014b).

We note that our present proposal leaves open the issue of the origin(s) of SNAs, be they a congenital result of hemispheric specializations, or acquired by culturally shaped spatial habits such as reading or finger counting (Fischer, 2008; Lindemann et al., 2011; Domahs et al., 2012; Fischer and Shaki, 2015; Rugani et al., 2015a,b). Assuming that processing number meaning is obligatorily accompanied by mapping it onto a spatial continuum, two issues remain to be addressed to account for a given SNA in a particular setting: The selection of the appropriate spatial dimension, and the directionality of mapping numbers along that dimension. We now present an idea of how these two steps are taken and describe recent evidence in support of this proposal.

First, *the spatial dimension selected for mapping of numbers reflects the stimulus and response features of the current task*. When lateralized response keys are provided to participants to measure the speed of their judgments, then most participants will align their number representations along the dimension indicated by these keys, be it horizontal, vertical, or radial. This is what the bulk of the literature has documented (as recently reviewed by Fischer and Shaki, 2014). In the absence of such response keys, when responses to numbers are required by making spatially directional arm, head, eye or whole-body movements, then the major directions or endpoints of those movements define the mapping dimension, again either using the horizontal (Fischer, 2003; Fischer et al., 2004; Loetscher et al., 2008; Shaki and Fischer, 2014a) or vertical dimension (Schwarz and Keus, 2004; Winter and Matlock, 2013). When spatially distinct responses to the numbers are required but no response dimension is prescribed, the resulting mapping of numbers onto space will be more varied across participants (Fischer and Campens, 2008). Finally, even when no spatially distinct responses are required, as for example in a simple detection task, the spatial mapping of centrally presented numbers will still emerge through lateralization of other stimuli, such as visually presented cues (Fischer et al., 2003; for a recent update, see Fischer and Knops, 2014).

Finally, once a dimension for the spatial mapping of numbers has been selected by the participant, their distribution along this dimension still remains to be decided. For this second step, we propose that *the orientation of the SNA is influenced by spatial experience*. This rule underlines the manifold of possible influences on the SNARC which are only beginning to be documented and studied. Living in a three-dimensional world, we are differentially sensitive to horizontal vs. vertical space. For example, as a result of the embodied nature of cognition, vertical distinctions are most salient and horizontal ones least salient (Fischer and Brugger, 2011), leading to faster acquisition of, and discrimination along, the vertical than the horizontal dimension (Franklin and Tversky, 1990). Similarly, the increasing strength of SNAs with age (Wood et al., 2008; Hoffmann et al., 2014) indicates that they may reflect accumulated spatial habits/experiences during life. An example are reading habits (see the contribution of Nuerk et al., 2015 to this research topic for a detailed description of mechanisms). Importantly, such life-long experiences are less powerful in determining the directionality of a SNAs compared to more recent experiences with numbers, as demonstrated in emerging training studies (e.g., Fischer, 2012) and by rapid alternations of SNAs between successive trials (Fischer et al., 2009).

In summary, the proposed two successive steps seem to capture a wide range of observations pertaining to the ubiquity of SNAs that have recently re-invigorated research into numerical cognition. We hope that the present proposal will guide further interest in the design of novel studies that aim to test specific predictions about the origin and strength of SNAs. For example, how can we identify the sequential nature of the mapping process? How shall we weight the contributions of previous experiences? Clearly, such questions identify numerical cognition as a convenient test-bed for the study of fundamental principles of cognition generally.

## Conflict of interest statement

The authors declare that the research was conducted in the absence of any commercial or financial relationships that could be construed as a potential conflict of interest.
